# Proton beam irradiation inhibits the migration of melanoma cells

**DOI:** 10.1371/journal.pone.0186002

**Published:** 2017-10-10

**Authors:** Katarzyna Jasińska-Konior, Katarzyna Pochylczuk, Elżbieta Czajka, Marta Michalik, Bożena Romanowska-Dixon, Jan Swakoń, Krystyna Urbańska, Martyna Elas

**Affiliations:** 1 Department of Biophysics, Faculty of Biochemistry, Biophysics and Biotechnology, Cracow, Poland; 2 Department of Cell Biology, Faculty of Biochemistry, Biophysics and Biotechnology, Cracow, Poland; 3 Department of Ophthalmology and Ophthalmic Oncology, Jagiellonian University Medical College, Cracow, Poland; 4 Institute of Nuclear Physics, PAS, Cracow, Poland; ENEA Centro Ricerche Casaccia, ITALY

## Abstract

**Purpose:**

In recent years experimental data have indicated that low-energy proton beam radiation might induce a difference in cellular migration in comparison to photons. We therefore set out to compare the effect of proton beam irradiation and X-rays on the survival and long-term migratory properties of two cell lines: uveal melanoma Mel270 and skin melanoma BLM.

**Materials and methods:**

Cells treated with either proton beam or X-rays were analyzed for their survival using clonogenic assay and MTT test. Long-term migratory properties were assessed with time-lapse monitoring of individual cell movements, wound test and transpore migration, while the expression of the related proteins was measured with western blot.

**Results:**

Exposure to proton beam and X-rays led to similar survival but the quality of the cell colonies was markedly different. More paraclones with a low proliferative activity and fewer highly-proliferative holoclones were found after proton beam irradiation in comparison to X-rays. At 20 or 40 days post-irradiation, migratory capacity was decreased more by proton beam than by X-rays. The beta-1-integrin level was decreased in Mel270 cells after both types of radiation, while vimentin, a marker of EMT, was increased in BLM cells only.

**Conclusions:**

We conclude that proton beam irradiation induced long-term inhibition of cellular motility, as well as changes in the level of beta-1 integrin and vimentin. If confirmed, the change in the quality, but not in the number of colonies after proton beam irradiation might favor tumor growth inhibition after fractionated proton therapy.

## Introduction

Proton beam radiation is used to treat malignancies because of its superior biophysical properties concerning dose deposition in tissues compared to photon radiation [1]. In contrast to the widely accepted view, that the two types of radiation exert similar biological effects in tissues, with the relative biological effectiveness of 1.1, several intriguing differences between low-energy proton beam and photon irradiated tumor cells have been reported. For example, homologous recombination was more significant for proton beam induced DNA damage [2]. High-LET proton beam irradiation caused cluster DNA damage with higher complexity with increasing LET [3], but low-LET proton beam caused similar DNA damage to photon irradiation [4]. Other differences were found in the level of the production of free radicals, cell cycle inhibition and apoptotic signaling [5]. In vitro treatment of tumor cells with a proton beam resulted in a higher percentage of apoptotic cells when compared to photon radiation [6]. Additionally, differences were observed in cell cycle regulation: a high-LET proton radiation induced a G2 phase arrest which was noticeably longer and harder to resolve in comparison to similar doses of photon radiation [7]. This was not seen for low-LET proton radiation [8].

Radiation may also affect the formation of metastasis, including cell detachment from the primary tumor, migration along the extra-cellular matrix (ECM), degradation of the basement membrane, and intravasation into the blood or lymphatic vessels [9]. Tumor cell-migration itself is a multistage process which depends on various factors such as proteinase activity [10,11], the cytoskeleton organization of the migrating cells [12] and adhesion to the ECM mediated by receptors such as integrins. Radiation may affect many of these steps, and a differential influence of proton and photon radiation has been suggested [5].

As proton beam therapy as well as radio-active plaque therapy are mainstays in the treatment of uveal melanoma, we wondered how these different approaches affected melanoma cells. We therefore studied the long-term effects of sublethal doses of proton beam irradiation and of photon treatment on the migratory properties of uveal melanoma and metastatic human melanoma skin cells. We tested cellular survival, motility and the level of β1-integrin and vimentin after proton beam and photon irradiation and showed that proton beam, but not photon irradiation, inhibited cellular rectilinear motility and changed heterogeneity of colonies. These effects were observed at long-term after treatment.

## Materials & methods

### Cell culture

We used Mel270, a primary human uveal melanoma cell line [13], and BLM, a cell line derived from the lung metastases of skin melanoma [14]. Both cell lines were cultured at 37°C, 5% CO_2_ in RPMI media (Sigma-Aldrich, St. Louis, MO), supplemented with 10% fetal bovine serum (Biological Industries, Cromwell, CT) and penicillin/streptomycin (Polpharma, Poland). The Mel270 cells were a gift from prof. M. Jager from Leiden University (The Netherlands) and BLM cells from Dr G.N.P. van Muijen, Department of Pathology, Radboud University Nijmegen Medical Centre, Nijmegen (The Netherlands). The cells were passaged at 70–80% of confluence every 5–6 days, so that the 4^th^ passage was at day 20 post-radiation and the 7^th^ passage was at day 40.

### Irradiation

Cells irradiations with X-rays and high energy protons were performed at the Institute of Nuclear Physics, Polish Academy of Sciences (IFJ PAN), Cracow, Poland. For X-ray irradiation Phillips MCN-323 tube at the voltage of 250 kVp and the dose rate of 1.8 Gy/min was applied. The beam filtered with 4 mm beryllium and additional 1 mm of brass. Dosimetry was performed using the PTW TM31013 ionization chamber with PTW UNIDOS electrometer, calibrated in the secondary standard laboratory at the Central Office of Measures in Warsaw, Poland. Cell cultures were irradiated in Eppendorf tubes placed on the surface of the PMMA phantom, Eppendorf's wall thickness was sufficient to compensate for the build-up effect. Proton beam irradiation took place at the Cyclotron Centre Bronowice at IFJ PAN. The 230 MeV proton beam produced at the IBA Proteus C-235 cyclotron [15] and degraded at the energy selector to the 70 MeV was delivered to the eye treatment room and mechanically formed using a set of scaterers and energy modulator at the eye irradiation unit. Fully modulated proton beam with energy 61 MeV (31.5 mm range in water) collimated to the 40 mm lateral diameter has been used for irradiation. During the irradiation the doses 1, 3, or 5 Gy have been delivered with dose rate of 1 Gy/min, 2 Gy/ min and 6.6 Gy/min, respectively. At the center of cell container position i.e. at the depths 15.8 mm of the SOBP the calculated Continues Slowing Down Approximation (CSDA) dose averaged LET_d_ was 2.8 keV/μm. Beam dosimetry was performed according to the TRS-398 protocol recommended by International Atomic Energy Agency [16] using a reference dosimeter consisting of a PTW TM31010 semiflex ionization chamber and a PTW UNIDOS Webline electrometer (PTW, Freiburg, Germany). The dosimeter set was calibrated at the IFJ PAN at Theratron 780 ^60^Co treatment unit. Cells were irradiated in the Eppendorf tubes positioned orthogonally to the direction of the proton beam using the dedicated phantom made out of PMMA. Cells were transported on ice between the facilities, including the untreated control.

### Clonogenic cell survival assay

The number of cells seeded into 6 cm diameter dishes was adjusted for each dose to achieve the optimal number of colonies after radiation. The experiment was performed three times and three replicate plates were seeded for each group in every repetition. For BLM and Mel270 cells alike, 100 (control), 300 (1 Gy), 500 (3 Gy) and 700 (5 Gy) cells were used in the case of proton beam radiation and the numbers of seeded cells for X-rays were 100 (control), 200 (1 Gy), 500 (3 Gy) and 700 cells (5 Gy). The plates were incubated for 2 weeks and then the cells were fixed and stained with Giemsa stain (Sigma-Aldrich, St. Louis, MO). The colonies formed were counted, with minimum 50 cells/colony, and PE (plating efficiency) and SF (surviving fraction) were evaluated. The colonies were divided into three groups according to their size determined as holo-, mero-, and paraclones, as described earlier [17]. The number of cells in colonies were: (i) 2500–6000 for holoclones, (ii) 500–2500 for meroclones and (iii) <500 for paraclones.

$$PE=\frac{Number\;of\;colonies\;counted}{Number\;of\;cells\;plated}\times 100$$ (1)

$$SF=\frac{PE\;of\;treated\;sample}{PE\;of\;control}\times 100$$ (2)

RBE (Relative Biological Effectiveness) was calculated as the ratio of the absorbed dose of reference radiation (X-rays) to the absorbed dose of radiation being researched (proton beam) which causes the same biological effect (37% of cells that survived treatment).

### MTT assay

The metabolic activity of Mel270 and BLM cells was measured with tetrazolium dye MTT (3-(4,5-dimethylthiazol-2-yl)-2,5-diphenyltetrazolium bromide) assay. The cells were seeded into 24-well plates (10^4^ cells per well), and cell numbers were determined each day during the first five days directly after irradiation and at 20 and 40 days post-treatment. Cells were supplemented with 10% of MTT (Sigma-Aldrich, St. Louis, MO) stock solution (0.5 mg/ml) and incubated for 2.5 hrs. The MTT formazan crystals that formed were dissolved in DMSO (Avantor, Poland) and methanol (Avantor, Poland) solution (1:1). Absorbance was measured at a wavelength of 560 nm with the Tecan GENios Plus plate reader (Tecan, Switzerland).

### Cell migration

Time-lapse monitoring of individual cell movements was used as an indicator of cellular migration properties. The individual trajectories of cells were assessed 20 days (4^th^ passage) and 40 days (7^th^ passage) after irradiation in both cell lines. Cells were plated at a density of 72 cells/mm^2^. After 48 hours the migration of cells was recorded at 37°C for 10h, at 10 min intervals. The trajectories of individual cells were evaluated from the changes in cell centroid location, as described previously [18]. For each cell, the following variables were determined [19]: (i) average speed of cell movement, i.e. the total length of cell trajectory/time of recording; (ii) the total length of cell displacement (μm), i.e. the distance from the starting point direct to the cell's final position. The value of CME (Coefficient of Movement Efficiency) was calculated as the ratio of the total cell displacement to the total length of cell trajectory. For each value, 50 cells were analyzed from 3 different wells.

### Wound healing assay

The cells were plated onto 6-well plates (2.5 x 10^4^ cells per well) at 20 (4^th^ passage) and 40 (7^th^ passage) days and the assay was performed on the third day after seeding. A wound (scratch) was made with a sterile tip. Pictures of the wound were taken at time point 0 h and 9 h. The wound area was analyzed using ImageJ v.1.43U (Wayne Rasband, National Institute of Health, USA) and the percentage of wound healing was calculated.

### Invasion assay

Cell invasion was assessed with Boyden Chambers (8.0 μm pore size, Falcon, NY, USA) in 24-well plates. Cells (10^4^) were put onto the upper surface of chambers. Chambers were incubated at 37°C for 48 h, at which time the number of cells at the bottom of the wells were counted and the percentage of cells that invaded through the membrane was established.

### Western blot

Cell monolayers were lysed in lysing buffer containing 1M Tris-HCl (pH 7.5), 3M NaCl, NP_4_O, distilled water, a protease inhibitor cocktail (Roche, Switzerland), PMSF and sodium orthovanadate. Cells were centrifuged at 13 000 RPM, 4°C. The amount of protein was measured using the Bradford assay [20] and stored at -80°C until used. Equal amounts of protein (20 μg) were run on Bolt® Bis-Tris Plus gels (Invitrogen, Thermo Fisher Scientific) and transferred to a nitrocellulose membrane using iBlot® Dry Blotting System (Invitrogen, Thermo Fisher Scientific). The membranes were blocked with 5% skim milk in a TBS buffer with 1% of Tween 20 for 1h and incubated with primary antibodies against vimentin (D21H3) (Cell Signaling Technology, MA, USA) and β1 integrin (Cell Signaling Technology, MA, USA) at 4°C overnight. Membranes were washed 3 times in TBS and incubated with suitable secondary antibodies and then washed 3 times in TBS. Signals were detected using LumiGLO® chemiluminescent substrate (Cell Signalling Technology, Danveers, MA).

### Statistical analysis

Statistical analysis was carried out using Statistica v12 (StatSoft. Inc.). Since we compared more than three experimental groups, significance was determined by one-way analysis of variance (ANOVA) after evaluation of homogeneity of variances with Levene’s Test. The differences were considered to be statistically significant at probability levels of p<0.05, p<0.01 and p<0.001.

## Results

### Cell survival after proton and photon irradiation

**Fig 1:**
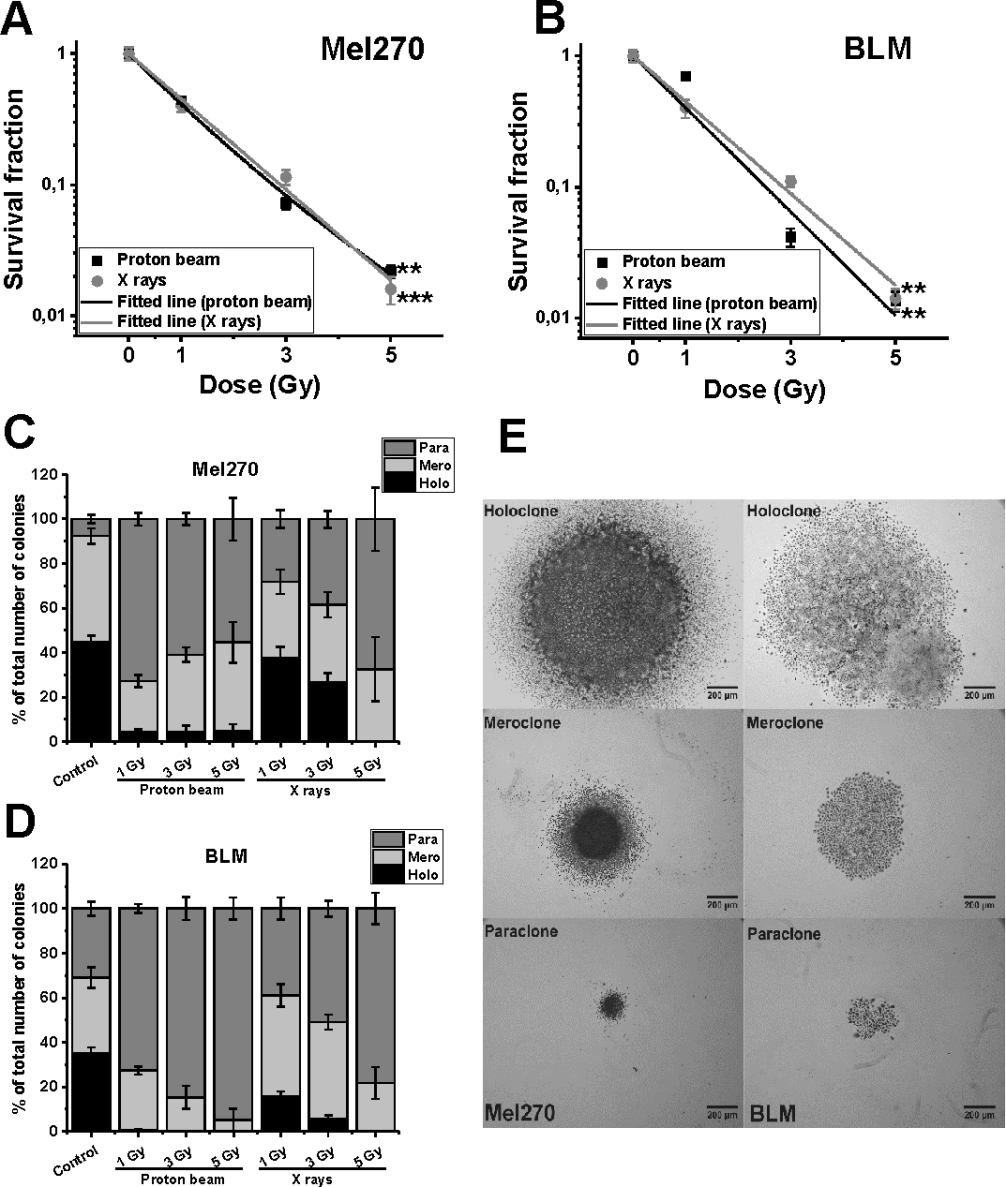
**Clonogenic assay of cell survival of Mel270 (A) and BLM (B) cells, treated with proton beam (■) or X rays (●).** Representative images of colonies are presented at S1 Fig. Cell were seeded immediately after radiation. Mean values with SEM, *p<0.05; **p<0.001. RBE values were determined from a linear-quadratic model and were 1.10 for Mel270, and 1.13 for BLM cells. (C–E) Three types of colonies formed by Mel270 (C) and BLM (D) cells in two weeks after irradiation with 1–5 Gy of proton beam or X-rays, determined as the percentage of the total number of colonies. Mel270 and BLM cells form three types of colonies described as holo-, mero- and paraclones (E). Holoclones are large, packed colonies displaying heterogeneity, which are believed to be derived from cancer initiating cells; meroclones are putatively derived from transit-amplifying cells and paraclones are loosely packed cells, derived from differentiated cells [17].

To compare the effects of radiation between uveal and skin melanoma cells, their radiosensitivity was determined using a clonogenic test after the two radiation qualities. A similar pattern of dose dependence of survival fraction (SF) in the clonogenic test was seen for both cell lines tested (Fig 1A and 1B). The Relative Biological Effectiveness (RBE) calculated for 37% of SF was very close, 1.1 for Mel270 and 1.13 for BLM. This is exactly the same value as that accepted for clinical proton radiotherapy [21].

To assess cell viability MTT assay was performed to evaluate the metabolic activity of cells directly after proton beam and X-ray radiation, as well as 20 and 40 days post-irradiation. The long-term time-points were the same as in the migration activity tests. Mel270 showed higher sensitivity to proton beam radiation than to X-rays in the first five days directly after treatment (S2 Fig). At the later time-points of 20 and 40 days the metabolic activity of cells was not affected (S3 Fig).

### Irradiation changed heterogeneity of cellular colonies

According to the literature [17] we divided them into three groups: holo-, mero-, and paraclones (Fig 1C–1E). The largest holoclones, containing between 2500–6000 cells, are described as displaying heterogeneity and as derived from cancer-initiating cells, middle sized meroclones (500–2500 cells) are probably derived from transit-amplifying cells and the smallest paraclones (<500 cells) are loosely packed cells, derived from differentiated cells [17]. As the growth time of these colonies was 14 days, we estimated the average doubling time for each type of colonies. For holoclones the doubling time was 26–29 h, meroclones 29–33 h, and for paraclones 38 h, which reflected substantial differences in their proliferative capacity.

Non-treated primary Mel270 cells formed similar numbers of holo-, and meroclones with a small number of paraclones (7.6%) (Fig 1C). In contrast, BLM cells developed a similar number of all clone types (Fig 1D). Both proton beam and X-rays reduced the number of holoclones in both cell lines substantially. However, proton beam caused a decrease in the number of holoclones from approx. 45% to 4% in Mel270 (all doses) and from 35% to almost none in BLM cells (0.38% for 1 Gy), forming paraclones instead. In contrast, X-ray irradiation resulted in considerable formation of holoclones after 1 and 3 Gy in Mel270 (from 45% to 34% and 24.6%, respectively) as well as in BLM cells (from 35% to 15.6% and 5.6%, respectively). The number of paraclones increased in a dose-dependent manner up to 67.5% for Mel270 and 78% for BLM cells. These results show the heterogeneity of both cell line populations and suggest that both types of irradiation shift the formation of colonies from holoclones (highly proliferative) to paraclones (less proliferative activity), even though the overall resulting surviving fraction, calculated from the total number of colonies, is similar for both types of radiation.

### Proton beam radiation inhibited the motility of cells and changed their direction of movement

**Fig 2:**
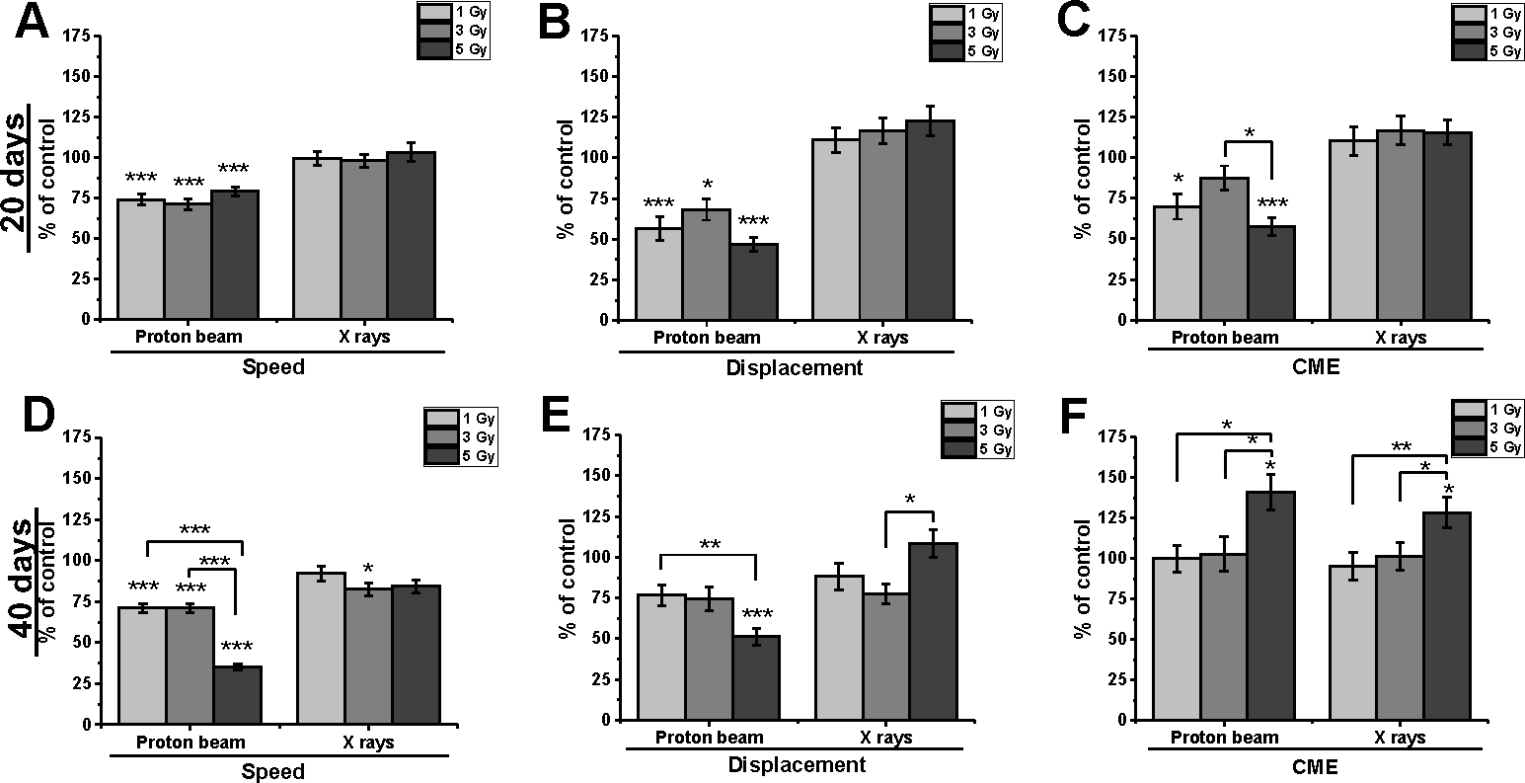
Cellular migration properties of Mel270 cells treated with proton beam radiation or X rays. Individual cell movements were evaluated at 20 days after irradiation (A, B, C) and at 40 days after irradiation (D, E, F) and three parameters were calculated: ‘Speed’, i.e. average speed of cell movement; ‘Displacement’, i.e. the total linear length of the cell displacement from the starting point (μm) and CME (coefficient of movement efficiency), i.e. the ratio of cell displacement to the cell trajectory length. Mean values presented as percent of control; *p<0.05, **p<0.01, ***p<0.001.

Long-term irradiation effects were compared for both radiation qualities in Mel270 and BLM cells. An analysis of Mel270 time-lapse recordings (S4 Fig) revealed inhibition of motility at 20 days after proton beam radiation (Fig 2), at reduced values of Speed (71–79%) and Displacement (47–68%). The CME (coefficient of movement efficiency) values were also reduced for all doses. Reduced CME suggests a change in the direction of movement. The lower the CME, the more cells move in circles rather than in a straight line. The motility of Mel270 cells, at 40 days after treatment with proton beam radiation, was inhibited to 71.2% of control, with a stronger effect after 5 Gy (Speed 35.3%; Displacement 52%), which translates into an increase in CME for 5 Gy. Therefore, the overall motility of uveal melanoma cells was inhibited at 40 days as well, but without the impact on the direction of cell movement.

**Fig 3:**
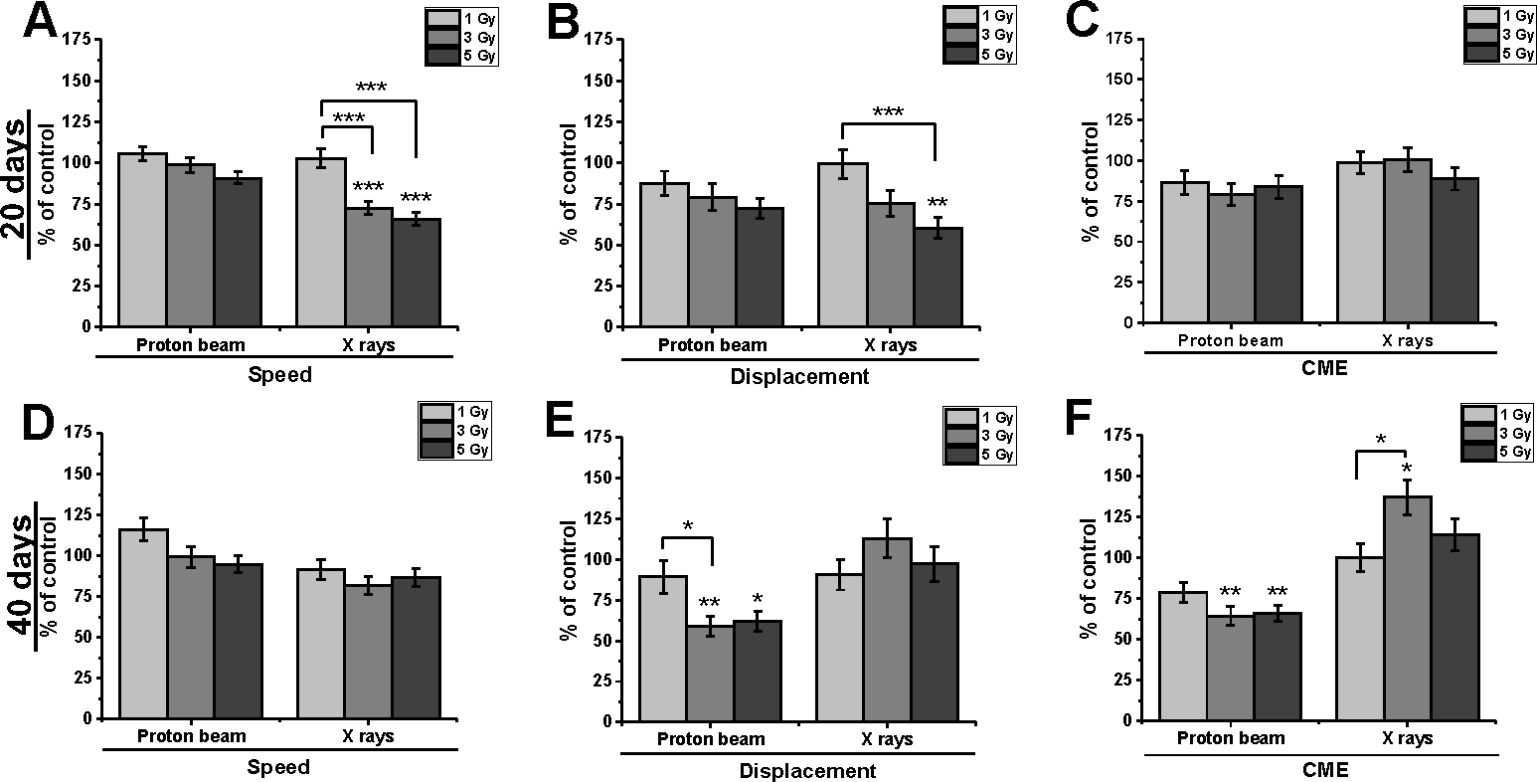
Cellular migration properties of BLM cells treated with proton beam radiation or X rays. Individual cell movements were evaluated at 20 days after irradiation (A, B, C) and at 40 days after irradiation (D, E, F) and were evaluated in terms of ‘Speed’, i.e. average speed of cell movement; ‘Displacement’, i.e. the total linear length of the cell displacement from the starting point (μm) and CME (coefficient of movement efficiency), i.e. the ratio of cell displacement to the cell trajectory length. Mean values presented as percent of control; *p<0.05, **p<0.01, ***p<0.001.

Irradiation of cutaneous melanoma BLM cells led to slightly different results (Fig 3). Following proton beam irradiation a decrease in Speed values was seen, with lower cell Displacement. This was reflected in CME, which decreased to 79–87% at 20 days and 64–79% at 40 days post-treatment and indicated less rectilinear movement. The direction of cell movement was not changed after X-rays.

In summary, proton beam irradiation inhibited motile activity in both cell lines. In the uveal melanoma cell movement was reduced, whereas in a metastatic cutaneous BLM cell line cell movement was more random.

### Both types of radiation slowed down wound closure

**Fig 4:**
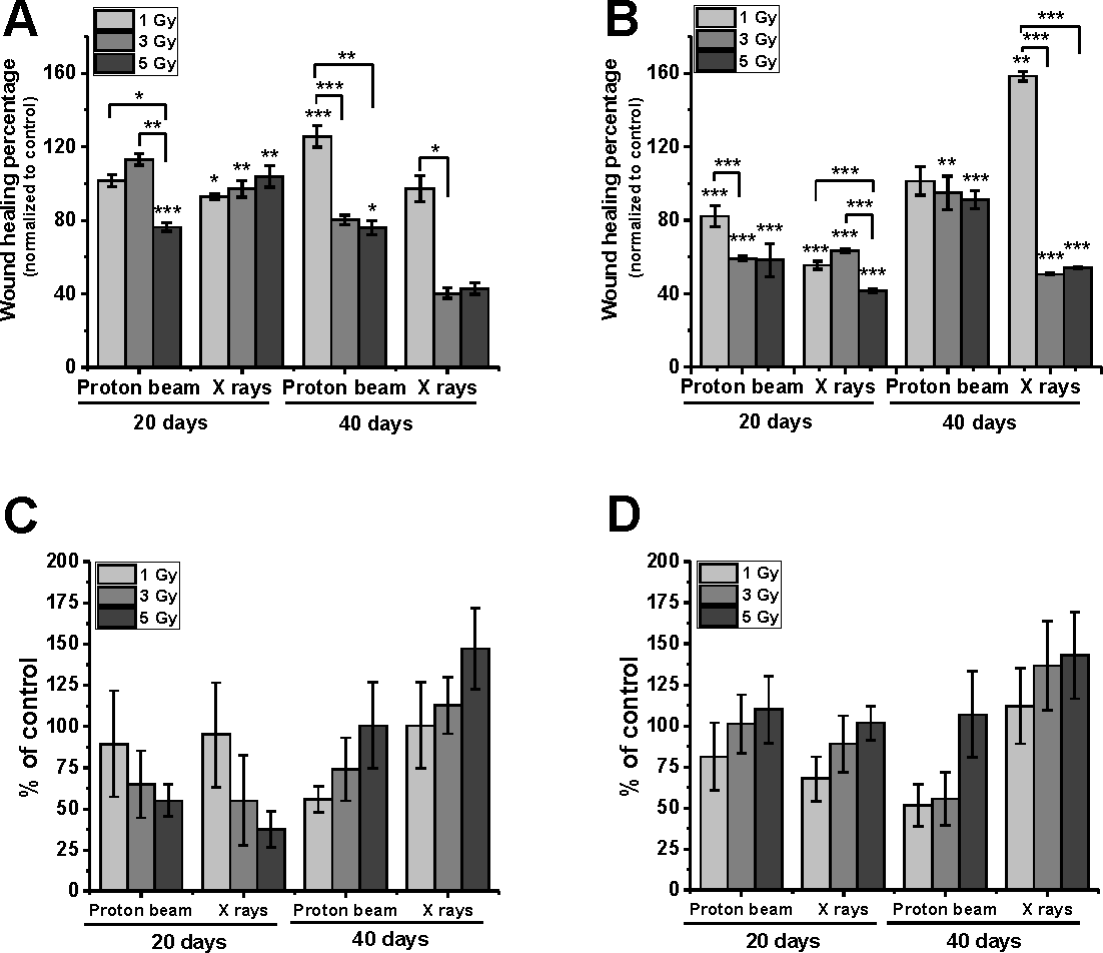
**Wound healing assay conducted for Mel270 (A) and BLM (B) cells, and cell invasion assay for Mel270 (C) and BLM (D).** Both assays were performed 20 and 40 days post treatment with proton beam or X rays. For wound healing test cell confluent monolayers were wounded with a pipette tip and images of the wound closure were taken after 9 hours of incubation. For each of two time-points 15–20 images were captured. Bars present the wound healing percentage normalized to control. *p<0.05; **p<0.01, ***p<0.001. Cell invasion was assessed by measuring the transpore migration with Boyden chamber assay (not coated, pore size 8 μm). Mean values in each group show the percentage of cells that migrated through the membrane in relation to control seeded in the well without a membrane. Values presented as the % of control. Mean ±SEM; *p<0.05.

Both types of radiation decreased the wound regrowth rate, although at different time scale in the two melanoma cell types. The inhibition of wound closure at 40–80% of control was observed in Mel270 cells at 40 days and in BLM cells at 20 days post-irradiation (Fig 4A and 4B). Some inhibition was also seen for BLM cells treated with 3 and 5 Gy of X-rays. What is more, the doubling time for the cell population of each experimental group was at least 30 hrs, therefore the assay results were not affected by proliferation.

### Cell invasion

The transmigration of cells through Boyden chambers (pore 8 μm) was related to the control seeded without membrane and presented as the percentage of the non-treated control cells in Fig 4C for Mel270 and in Fig 4D for BLM. In both proton beam treated cell lines, a decrease in transmigration was seen, especially at 40 days post-irradiation. In contrast, a lower number of X-ray irradiated cells was only observed at 20 days for Mel270 cells, but the numbers were not statistically significant.

### Irradiation decreased the level of β1-integrin

**Fig 5:**
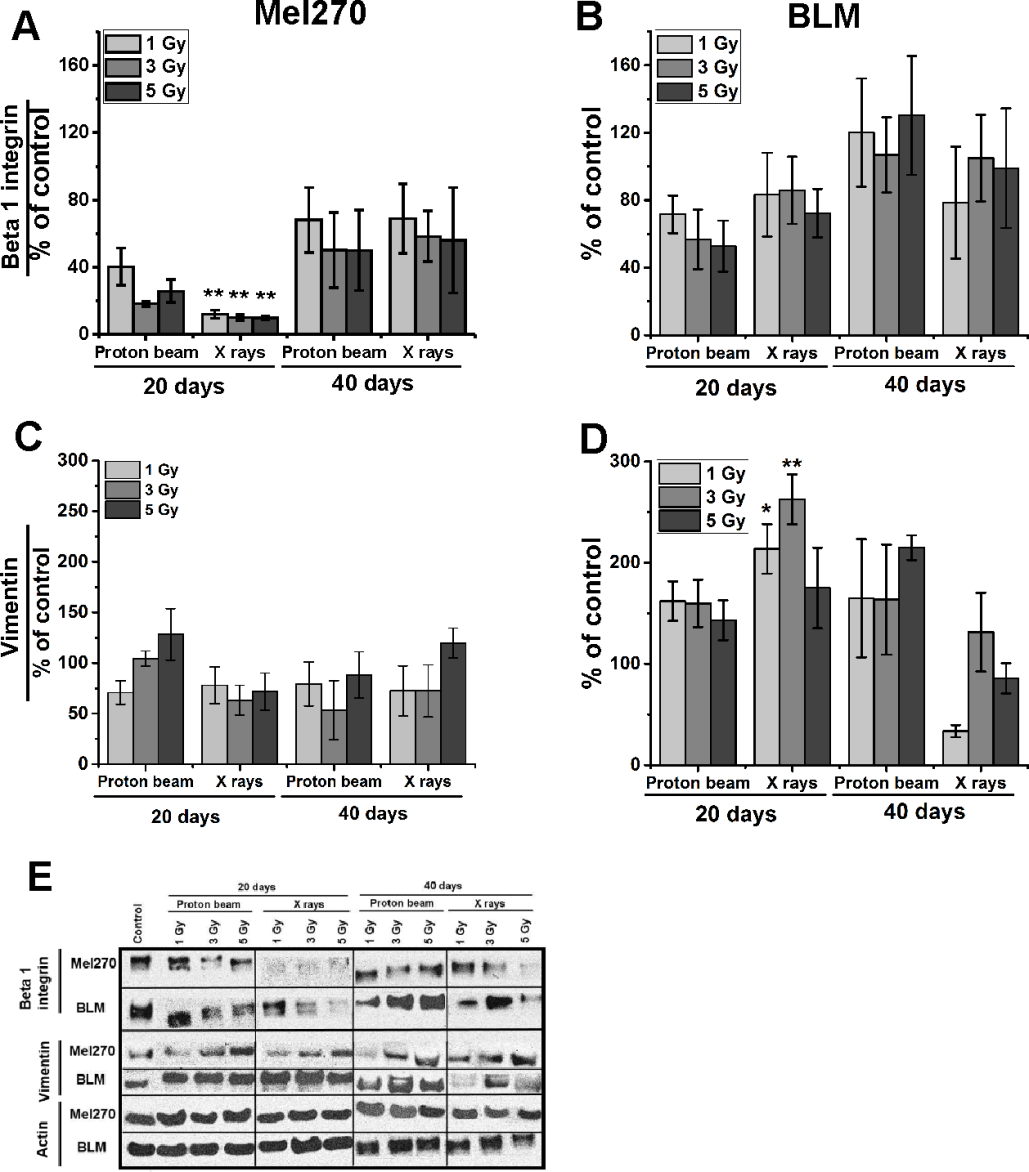
**Integrin β1 (A, B) and vimentin (C, D) protein expression assessed with Western Blot in Mel270 (A, C) and BLM (B, D) cells treated with different doses (1, 3, 5 Gy) of proton beam or X rays.** Cells were lysed 20 and 40 days after irradiation. (E) 20 μg of protein was applied per well. Control of untreated cells was set to a 100%. Chemiluminescent evaluation of 3 independent Western blots of cell lysates was shown as a mean of the percentage of the control and SEM. *p<0.05; **p<0.01; ***p<0.001.

We performed WB analysis for integrin β1, which is involved in metastasizing and known to decline after irradiation. Both types of irradiation strongly suppressed integrin β1 in Mel270 cells (Fig 5A) at 20 days post-treatment, the values ranged between 18%–40% and 10%–12% for proton beam and X-rays respectively. At 40 days the cells showed an increase in their β1-integrin level, although it still remained below the control level (50%-69%). In contrast, in BLM cells (Fig 5B) the lowering of β1-integrin was seen only after 5 Gy at 20 days.

### BLM cells show a higher expression of vimentin after irradiation

No significant differences in vimentin, one of the markers for epithelial-mesenchymal transition (EMT), were found in Mel270 cells (Fig 5C) after irradiation. However, BLM cells (Fig 5D) treated with a proton beam displayed an increase in the protein level of between 143 and 162% at 20 days and between 163 and 214% at 40 days after irradiation. The increase was also observed after X-rays at 20 days following treatment, especially in the case of 3 Gy (263%). Nevertheless, at 40 days the vimentin expression decreased approximately to the control level.

## Discussion

The comparison of the effects of two radiation qualities on melanoma cells showed distinct differences in the colonies generated as well as long-term migratory properties. Despite the fact that the two melanoma lines tested were of a different origin (uveal and cutaneous), in both of them the proton beam, but not photon radiation, caused the inhibition of actively proliferating cells and long-term motility inhibition. As we compared long-term effects, we used sublethal doses of two types of radiation. These sublethal doses may be significant in fractionated therapy or in combination therapies involving radiation treatment. What is more, we have to consider margin of radiation during treatment. In spite of restrained area of the margin in the case of proton beam irradiation, the tumor cells located within may contribute to formation of metastatic lesions.

### Low-LET proton beam irradiation inhibited actively proliferating cells

Our study showed that at 2 weeks post-treatment, overall clonogenic survival of both melanoma cells *in vitro* (Fig 1A and 1B) treated with either X-rays or proton beam irradiation is very similar with fairly close RBE values. However, when we take into consideration the subpopulations of clones featured (Fig 1) we observed a shift in the colonies formed after irradiation. This was manifested by a dramatic increase in the number of less active clones (paraclones). These cells were physically present in the irradiated population and yet may have lost their capacity for sustained proliferation. Such a state may be called a reproductive death, i.e. a cell may be physically intact but has lost its ability to divide. Both kinds of irradiation, but especially proton beam irradiation drastically diminished the number of holoclones (containing cancer stem-like cells [17]) in both cell lines. Such a drastic decrease in the number of holoclones, containing actively proliferating cells, with a concomitant increase in less active paraclones might have a profound effect *in vivo*. Consequently, fractionated proton beam radiation may result in a stronger inhibition of the tumor growth by decreasing actively the proliferating cell population. This hypothesis requires further investigation.

It has to be pointed out that the cells studied were not synchronized in the cell cycle, so the results observed are averaged over all cells in the population. What is more, we observed highly heterogeneous cell populations in both cell lines.

Melanoma metastatic cell line HTB140 response to proton beam irradiation was intensively studied [22,23]. It was shown that in comparison to gamma-rays, proton beam radiation induced more apoptotic cells, for doses ranging from 8 to 24 Gy [23]. In our study, we have focused on sublethal doses of radiation 1–5 Gy and observed slightly lower metabolic activity and decreased number of highly proliferative clones (S2 Fig and Fig 1).

Relative biological effectiveness of proton beam was widely studied depending on such factors as cell sensitivity, dose, LET, initial energy of the beam or the depth in SOBP [21,24,25]. RBE increases linearly with LET [26,27] and with α coefficient and decreases with increasing (α/β)_photons_ [28]. RBE values vary from 1.1 to 1.7 for 2 Gy per fraction [29,30] and can reach even 2.84 at the distal end of SOBP for very radioresistant cells [31]. Nevertheless usually in vivo studies show RBE at mid-SOBP approximately 1.1 ranging from 0.7 to 1.6 [27]. Such increase in RBE is significant, for example, application of variable RBE resulted in an increase of RBE weighted dose in the SOBP plateau by approximately 18% for both normal and tumor human cells [32]. Several models for predicting the RBE for proton beam were developed [24,33,34] and many authors postulate using RBE-weighted proton beam modulation, or LET-painting in the clinic [26,30,32,35].

### Low-LET proton beam irradiation inhibited cellular motility

At both long-term time-points an effect on cellular motility was seen. In Mel270 cells we observed a major influence on the direction of movement, which was also paralleled in the decreased ability of Mel270 cells to invade. In BLM cells the direction of cellular movement was inhibited by the proton beam at 40 days, which also was accompanied by numerous filopodia generated by the cells. In contrast to Zheng et al. [36] presenting the stimulating effect on the migratory and invasive potential of tongue squamous cell carcinoma at only 24 hours post radiation, we have not detected an increase in the motility of melanoma cells treated with X-rays at long term after irradiation.

Despite the inhibited motility, cell invasion ability was only slightly, or not at all affected by radiation, therefore it could be supposed that cell elasticity in cell migration and invasion plays a role. However, in our experimental setup, we did not use any chemoattractant in the transmigration test and only cells that went through the transpore and migrated to the well were counted. There may have been cells that were attached to the other side of the membrane, so we may have underestimated the number of cells.

Another evidence supporting the notion of proton beam radiation inhibiting cellular migration was shown in the studies, where proton beam irradiation of tumor cells and normal primary human lens cells resulted in down-regulation of MMP-2 and MMP-9 [37,38], therefore suggesting suppression of the cell migration. In contrast, photons led to the up-regulation of MMP-2 as well as of MMP-9 [37]. Hence the proton beam was thought to restrain cell migration while photons may have stimulated it.

### Suspected switch of the phenotype

Another interesting observation is a dramatic loss of β1 integrin in Mel270 cells at only 20 days post-radiation. Considering the absence of substantial changes in cell morphology one might speculate that the loss of β1 integrin may be compensated for with an increase in a different integrin. On the other hand, we did not see any changes in vimentin level in Mel270, which may indicate that the cells did not undergo an EMT after irradiation. Radiation-induced EMT was reported for example for colorectal cells [39]. Therefore, we postulate that Mel270 did not lose their epithelial phenotype as they display a strong connection with neighboring cells and EMT alter the integrity of cell-cell junctions, which results in the loss of contact between cells. In BLM cells, however, we observed only a slight loss of β1 integrin at 20 days after proton beam with an increase of vimentin level, which may suggest some shift towards EMT phenotype. Nevertheless, we need to consider the fact that these cell lines are of a different origin. BLM cells are derived from lung metastasis of cutaneous melanoma, therefore they have already gone through an EMT process, which is associated with an increase in vimentin level.

Switching phenotypes between epithelial and mesenchymal was recently reported in skin melanoma [40]. It was observed that melanoma cells of both proliferative and invasive populations were able to start a tumor *in vivo*, although the latter ones took a much longer time. However, in the end the tumors were comparable and it was suggested that the cells could switch phenotypes in both directions [41]. Despite the fact, that the proliferating population of cells may be the major contributor to the growth of a tumor, the phenotype switching mechanism may be used to evade growth arrest, therefore to acquire resistance, as in the case of NSCLC, where the authors observed a high level of vimentin and also ZEB1 and AXL1 [42]. In this context, our results might be interpreted in such a way that sublethal doses of radiation lead to switching, or shifting of the phenotype towards more mesenchymal, as we observed (i) phenotypic heterogeneity, (ii) changes in cell migration and direction of movement, (iii) differences in β1 integrin and vimentin levels. For further investigation EMT- associated signaling pathways should be explored, especially the transcription factors regulating the activity of the E-cadherin promoter and E-cadherin repressors and therefore the activity of the beta-catenin/TCF4 complex, MAP/ERK and JAK/STAT3 pathways [40].

### Further studies directions

Nevertheless we need to be aware of the complexity of the possible mechanisms underlying the differences in response of melanoma cells to proton beam and photon irradiation. Apart from EMT connected factors, many pathways and processes may be engaged in post-radiation changes in melanoma cells. As cell migration requires a coordinated adhesion contacts between cells as well as cell-ECM (extracellular matrix) interactions, there is a variety of intracellular and extracellular proteins such as integrins, cadherins and catenins, may have been affected [43]. Another aspects are the signaling pathways such as MAPK and WNT. MAPK pathway covers many kinase modules that convey extracellular signals to proteins controlling essential cellular processes such as cell growth, proliferation, differentiation, migration and apoptosis [44,45] and WNT signaling pathway is responsible for cell proliferation, migration and polarization [46]. Worth pursuing are the mechanisms of DNA damage repair, as it was already shown that homologous recombination is more important in repairing proton beam induced DNA damage [2] and that at the distal end of SOBP an increased complexity of DNA lesions and slower repair kinetics was observed [4]. Even though the direct consequences of proton beam radiation are being better understood each year, very little is known about its long term effects. Epigenetic changes may be responsible for maintaining post-radiation phenotype [47], and the presence of cancer stem cells may impact the post-radiation long-term response [48]. Therefore in order to fully explain the mechanisms behind of the difference between proton beam and photon radiation on melanoma cells further studies are required.

### Clinical relevance

As metastasis is the main reason of mortality of patients with both skin and uveal melanoma, any treatment inhibiting migratory properties of cells would be of benefit in the clinic. Proton beam therapy is used for treatment of uveal melanoma since 1975 and its results are comparable to brachytherapy, with the mean local control over 95%, and rate of complications 7.7% [49,50]. It may be speculated that if proton beam therapy indeed inhibits metastatic properties of cells, higher survival of uveal melanoma patients treated with proton beam might be seen, at least in the long term. However, it is not possible from the data presented in the literature, to conclude unequivocally whether such difference exists. Lane et al. point out in their recent analysis of a large cohort of UM patients treated with proton beam that 25-year cumulative UM-related mortality was approximately 30% for proton beam therapy and 50% after enucleation. For ^125^I brachytherapy, the COMS study report 12-year mortality for older patients with large tumors also at 30% [51].

On the other hand, one may argue that the effect of radiation on the cellular migratory properties is exerted at the time of treatment, when micrometastases are already present [52], and therefore the overall survival might not be affected. Perhaps some more light onto the role the migratory properties of uveal melanoma cells will in the future come from studies showing the mortality dependence on the genetic status of the tumor [53] and from a better understanding of the development of UM metastases [54].

## Conclusions

Our results indicate that there are several distinct differences between the effect of proton beam irradiation and X-rays on the survival mechanisms and migratory properties of melanoma cells. Proton beam radiation inhibited cellular rectilinear motility and decreased invasive potential in comparison to X-rays. β1 integrin level was decreased after both types of radiation in uveal melanoma cells, and the level of vimentin increased in BLM, cutaneous melanoma cells. An important observation is the change towards a less proliferative type of colonies generated after irradiation with a proton beam. If confirmed in an *in vivo* setting, this might have profound implications for the increased efficacy of fractionated proton beam radiotherapy.

## Supporting information

S1 Fig Representative images of colonies formed by Mel270 and BLM cells. The number of seeded cells is shown in the upper right corner of each plate photo.
(TIF)

S2 Fig **MTT test showing metabolic activity of Mel270 cells (A, B) and BLM cell line (C, D)**. Metabolic activity was estimated during the first five days directly after treatment (A, D) with proton beam or X rays expressed as percent of control for each day. Mean values, with SEM, #p<0.05; *p<0.01; **p<0.001.
(TIF)

S3 Fig **MTT test showing metabolic activity of Mel270 (A) and BLM (B) cell line after 20 days and 40 days post irradiation**. It was expressed for each day as percent of control. Mean values, with SEM, #p<0.05; *p<0.01; **p<0.001.
(TIF)

S4 Fig Individual trajectories of 50 non-dividing Mel270 cells expressed as circular diagrams. Single line represent a single cell trajectory with initial point of each trajectory set at the 0 point of the diagram. Cells were seeded 20 days after irradiation with proton beam or X-rays. Cell movement was recorded for 10 hrs, with 10 min intervals. A representative transmitted light image of the cells is to the right (magnification 200x).
(TIF)
