# Supplementary figures and images for: Proton beam irradiation inhibits the migration of melanoma cells
Source: PLoS One. 2017 Oct 10;12(10):e0186002. doi: 10.1371/journal.pone.0186002 (PMC5634624; doi:10.1371/journal.pone.0186002)

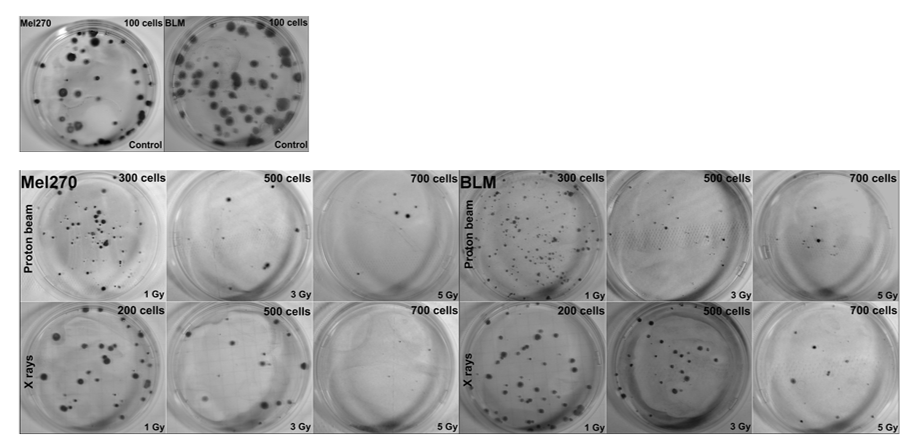

Supplement: S1 Fig — The number of seeded cells is shown in the upper right corner of each plate photo. (TIF) [file pone.0186002.s001.tif]

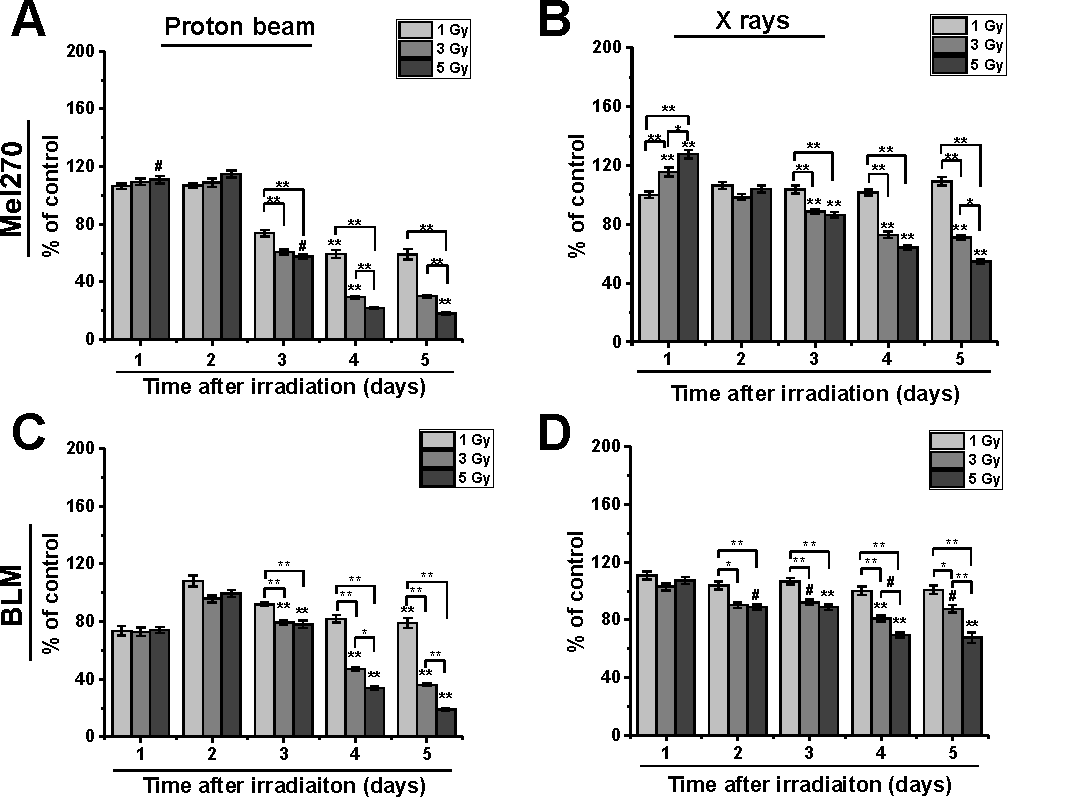

Supplement: S2 Fig — MTT test showing metabolic activity of Mel270 cells (A, B) and BLM cell line (C, D). Metabolic activity was estimated during the first five days directly after treatment (A, D) with proton beam or X rays expressed as percent of control for each day. Mean values, with SEM, #p<0.05; *p<0.01; **p<0.001. (TIF) [file pone.0186002.s002.tif]

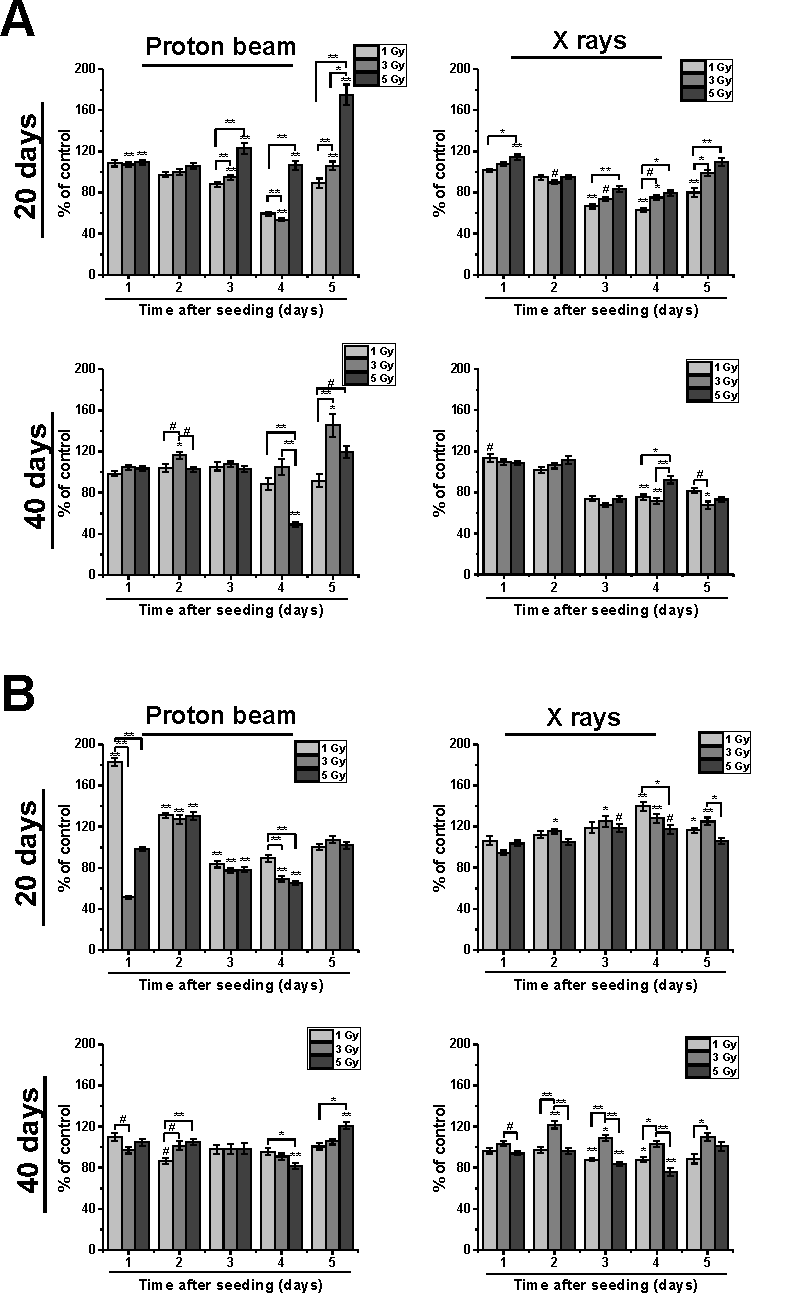

Supplement: S3 Fig — MTT test showing metabolic activity of Mel270 (A) and BLM (B) cell line after 20 days and 40 days post irradiation. It was expressed for each day as percent of control. Mean values, with SEM, #p<0.05; *p<0.01; **p<0.001. (TIF) [file pone.0186002.s003.tif]

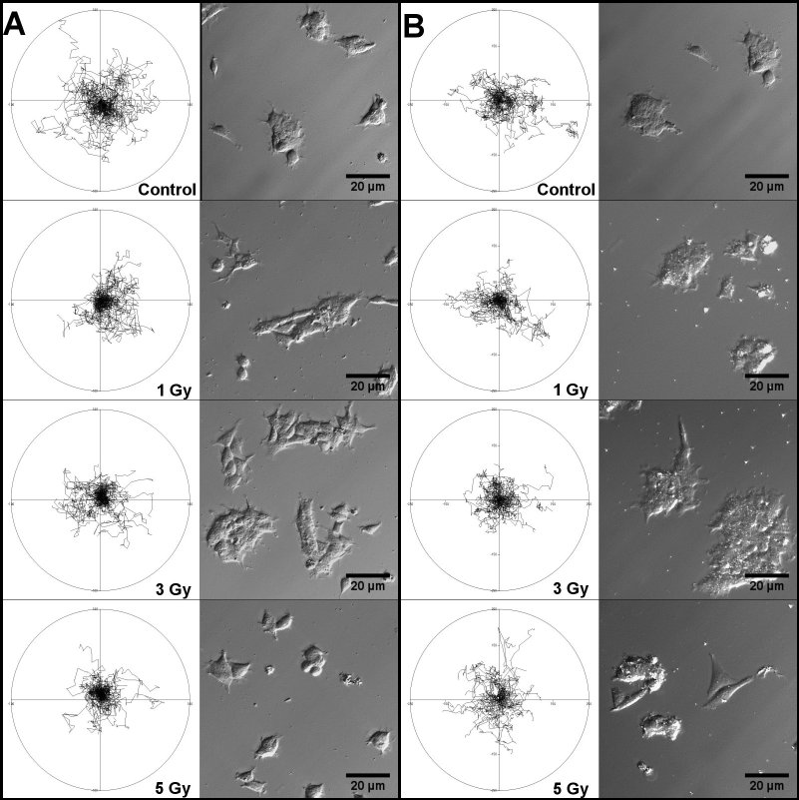

Supplement: S4 Fig — Single line represent a single cell trajectory with initial point of each trajectory set at the 0 point of the diagram. Cells were seeded 20 days after irradiation with proton beam or X-rays. Cell movement was recorded for 10 hrs, with 10 min intervals. A representative transmitted light image of the cells is to the right (magnification 200x). (TIF) [file pone.0186002.s004.tif]
